# Whole-Genome Sequences of Six *Salmonella enterica* Serovar Bovismorbificans Isolates Associated with a 2011 Multistate Hummus-Borne Outbreak

**DOI:** 10.1128/genomeA.01239-13

**Published:** 2014-03-06

**Authors:** Gopal Gopinath, Junia Jean-Gilles Beaubrun, Chris Grim, Morris Blaylock, Reginald Blackwell, Sosina Merid, Alpha Diallo, Darcy Hanes

**Affiliations:** a U.S. Food and Drug Administration, Center for Food Safety and Applied Nutrition, Office of Applied Research and Safety Assessment, Division of Virulence Assessment, Laurel, Maryland, USA; b District of Columbia Public Health Laboratory, Department of Forensic Sciences, Washington, DC, USA

## Abstract

We present six draft genome sequences of *Salmonella enterica* serovar Bovismorbificans from isolates associated with the 2011 hummus-borne multistate outbreak. All six genome sequences indicate the presence of two plasmids, one of which demonstrates similarity to the 93-kb pSLT2 IncF-type plasmid of *Salmonella enterica* serovar Typhimurium.

## GENOME ANNOUNCEMENT

There are more than 40,000 cases of salmonellosis reported in the United States each year (see the CDC reports of *Salmonella* outbreak investigations [http://www.cdc.gov/salmonella/outbreaks.html]). In August through November of 2011, sesame seed paste (tahini) and hummus containing a rare serotype of *Salmonella enterica* caused illness in 23 people in 7 states and the District of Columbia (see the CDC report on *Salmonella* pathogens and protocols [http://www.cdc.gov/pulsenet/pathogens/salmonella.html]) (1). Reported cases were largely concentrated in the Mid-Atlantic region, with eight in Washington, DC, seven in Maryland, three in Virginia, and one each in Delaware and New Jersey. Three cases were also reported outside this region—one in California, one in Michigan, and one in New Hampshire (see the CDC report on *Salmonella* pathogens and protocols and reference 1). The *Salmonella enterica* serovar Bovismorbificans strains isolated from the 2011 hummus-associated outbreak demonstrated a pulsed-field gel electrophoresis (PFGE) pattern that was distinctly different from those of other isolates of serovar Bovismorbificans recorded in the Pulse Net Database (see the CDC reports and reference 1). In this report, we announce the availability of six draft genome sequences of *Salmonella* serovar Bovismorbificans from isolates associated with this outbreak: three isolates (SAL609, 610, and 615) from patient stool samples, two from hummus samples (SAL616 and 617) from the District of Columbia Public Health Laboratory, and one clinical isolate (SAL682) from the Michigan Department of Health.

Genomic DNA from each strain was extracted from overnight cultures using QIAcube (Qiagen). The DNA library was constructed according to the Illumina recommended protocol for Nextera XT DNA sample prep and sequenced using Illumina Miseq (San Diego, CA). CLC bio software version 6.0.1 (Germantown, MD) was used for the trimming and *de novo* assembly of the paired-end reads to 100 contigs. The genomes were annotated by use of the RAST server (2).

The lengths and the GC contents of the draft whole-genome assemblies of the strains are as follows: SAL609, 4,896,135 bases and 52.1%; SAL610, 4,856,095 bases and 52.2%; SAL615, 4,845,484 bases and 52.1%; SAL616, 4,863,068 bases and 52.1%; SAL617, 4,870,084 bases and 52.1%; and SAL682 version 02, 4,843,320 bases and 52.2%. All six genomes indicated the presence of two plasmids, one of which demonstrates similarity to the 93-kb pSLT2 IncF-type plasmid of *S. enterica* serovar Typhimurium. In addition to this virulence plasmid, an integration and conjugative element (ICE) similar to ICESb1 found in *Salmonella bongori* (3) has been identified in these outbreak strains. Unlike in other *Salmonella* serovars, in this serovar some phages and type III, IV, and VI secretion systems were observed. A detailed analysis of these isolates of Bovismorbificans from a recent outbreak will shed light on the diversity and distribution of factors contributing to virulence mostly obtained by horizontal transfer.

### Nucleotide sequence accession numbers.

The draft genome sequence accession numbers for these six *Salmonella enterica* serovar Bovismorbificans strains as deposited in NCBI are AZKW00000000, AZKX00000000, AZKY00000000, AZKZ00000000, AZLA00000000, and AZKQ00000000 (the version described here is version 02, AZKQ02000000).
